# Comprehensive UAV and ground data for typical semiarid sites in the midstream of the Heihe River Basin

**DOI:** 10.1038/s41597-026-07151-0

**Published:** 2026-04-01

**Authors:** Ji Zhou, Ziwei Wang, Shaomin Liu, Mingsong Li, Jin Ma, Lingxuan Meng, Nanjie Feng

**Affiliations:** 1 School of Resources and Environment, University of Electronic Science and Technology of China, Chengdu, 611731 China; 2 State Key Laboratory of Earth Surface Processes and Hazards Risk Governance (ESPHR), Faculty of Geographical Science, Beijing Normal University, Beijing, 100875 China

**Keywords:** Hydrology, Agroecology

## Abstract

Understanding land surface processes in arid and semiarid environments is crucial for ecosystem dynamics and water management. This data descriptor presents a comprehensive dataset collected during the MUlti-Scale Observation Experiment on land Surface temperature using UAV remote sensing (MUSOES-UAV). Acquired from June to October 2020 at typical semiarid sites in the midstream of the Heihe River Basin, China, the dataset includes high-resolution thermal infrared (TIR) and multispectral images from a UAV. The TIR data were corrected for temperature drift, while the multispectral images underwent radiometric relative normalization to ensure data consistency. Concurrently, ground-based observations were collected from TIR radiometers and automatic weather stations. The final dataset consists of TIR brightness temperature mosaics, multispectral mosaics, and normalized difference vegetation index (NDVI) maps, complemented by the ground-based measurements. This multi-scale dataset is a valuable resource for monitoring environmental changes and provides a foundational basis for developing and validating algorithms for UAV remote sensing applications.

## Background & Summary

Arid and semiarid regions face severe ecological challenges due to water scarcity and sparse vegetation^1–3^. While ecological monitoring and vegetation restoration are crucial for managing these fragile environments^4–8^, a major constraint is the lack of targeted, high-resolution, multi-temporal datasets. Traditional observational approaches have notable limitations in capturing fine-scale thermal and spectral dynamics^9–11^. Satellite remote sensing (e.g., MODIS, Landsat-9) provides broad spatial coverage but lacks the spatial granularity required for detailed ecological studies and is susceptible to cloud cover^12–16^. Manned aerial remote sensing offers high resolution but is constrained by prohibitively high operational costs and inflexible workflows^17,18^. Conversely, ground-based *in situ* measurements yield high accuracy but cannot provide continuous large-scale spatial observations due to geographical and resource constraints^19^. To overcome these limitations and bridge the gap between coarse satellite imagery and sparse ground observations, Unmanned Aerial Vehicles (UAVs) have emerged as an optimal, flexible platform for high-resolution data collection. UAVs can operate at low altitudes, capturing high-resolution thermal and multispectral imagery, enabling detailed monitoring of surface temperature variations and vegetation spectral characteristics^20–23^. Their high maneuverability allows flexible mission designs in complex terrains, while their ability to bypass cloud cover facilitates all-weather observation. These capabilities position UAV remote sensing as a highly effective tool for monitoring thermal environments and spectral dynamics in arid and semiarid regions, offering significant potential to advance ecological research and resource management in these challenging landscapes^24^.

Here, we present a set of UAV-based remote sensing datasets collected during the Multi-Scale Observation Experiment on land Surface temperature using UAV remote sensing (MUSOES-UAV), formally conducted from June to October 2020 in the midstream regions of the Heihe River Basin^25,26^. Although preliminary UAV-based observations were also conducted in 2019 in the same region as part of an initial experimental phase, the quality and completeness of the data collected during that period were limited due to evolving instrumentation and methodology. Therefore, the 2020 datasets presented in this paper provide higher-quality and more comprehensive observations, which offer improved reliability for further analysis. The observation areas included three representative field sites: the Daman site (oasis), the Huazhaizi site (desert), and the Wetland site (wetland). Each site covered a flight area of approximately 2.9–4.3 km^2^, designed to match the spatial resolution of satellite observations, particularly MODIS, while also encompassing the source area of eddy covariance observations. These datasets provide monthly thermal infrared (TIR) data (i.e., brightness temperature, BT), multispectral data, and normalized difference vegetation index (NDVI) data for each site, as well as complementary ground-based observations, focusing on the vegetation growing season within the experimental region. These datasets underwent rigorous post-processing to ensure high-quality and reliability. They offer high-resolution characterizations of the thermal and spectral properties of typical land surface features in arid and semiarid regions, bridging satellite observations with ground-based measurements to form a multi-scale observation network. This paper details the experiment and the data acquisition and processing methods. These datasets provide fine-scale insights into the ecological dynamics of arid and semiarid regions, contributing to ecosystem conservation while supporting the coordinated development of agriculture, ecology, and economy in these areas. Thanks to the relatively raw nature of the provided datasets, researchers are encouraged to freely develop various algorithms and models, fostering innovation in UAV remote sensing theory and methods.

While the spatial coverage of the UAV observations in this dataset is inherently localized compared to basin-scale satellite imagery, it provides critical ultra-high-resolution spatial details. Users should note that this dataset is not intended for direct, large-scale regional generalizations. Rather, it is specifically designed to bridge the scale gap between ground-based point measurements and coarse satellite pixels. Therefore, it is highly valuable for micro-ecological studies, precision agriculture, and serving as a robust testbed for the validation and downscaling of satellite-derived products in heterogeneous arid and semi-arid landscapes.

## Methods

### Study sites and scientific objectives

#### Study sites description

With a total area of about 143,000 square kilometers, the Heihe River Basin ranks as the second largest endorheic river basin in China, and is roughly located between 97.1°–102.0° E and 37.7°–42.7° N^27^. Situated in the arid region of western China, the basin primarily relies on cryospheric water sources from its upstream areas. The study area locates in the midstream section of the Heihe River Basin, within Zhangye City, Gansu Province. This region represents a typical oasis-desert system, covering a total area of approximately 29,700 square kilometers and encompassing a diverse range of land cover types, including farmland, wetlands, deserts, shelterbelt, orchard, and residential areas. The elevation of the midstream region generally decreases from south to north, ranging from approximately 1,000 to 3,000 m^28,29^. The area is characterized by an arid climate, limited precipitation, and high solar radiation, with an annual average precipitation of about 100–250 mm^30^. Because of its distinct and highly heterogeneous geographical and climatic conditions, the region has been the focus of numerous studies including hydrology, meteorology, and ecological restoration, among others^31–34^.

**Fig. 1:**
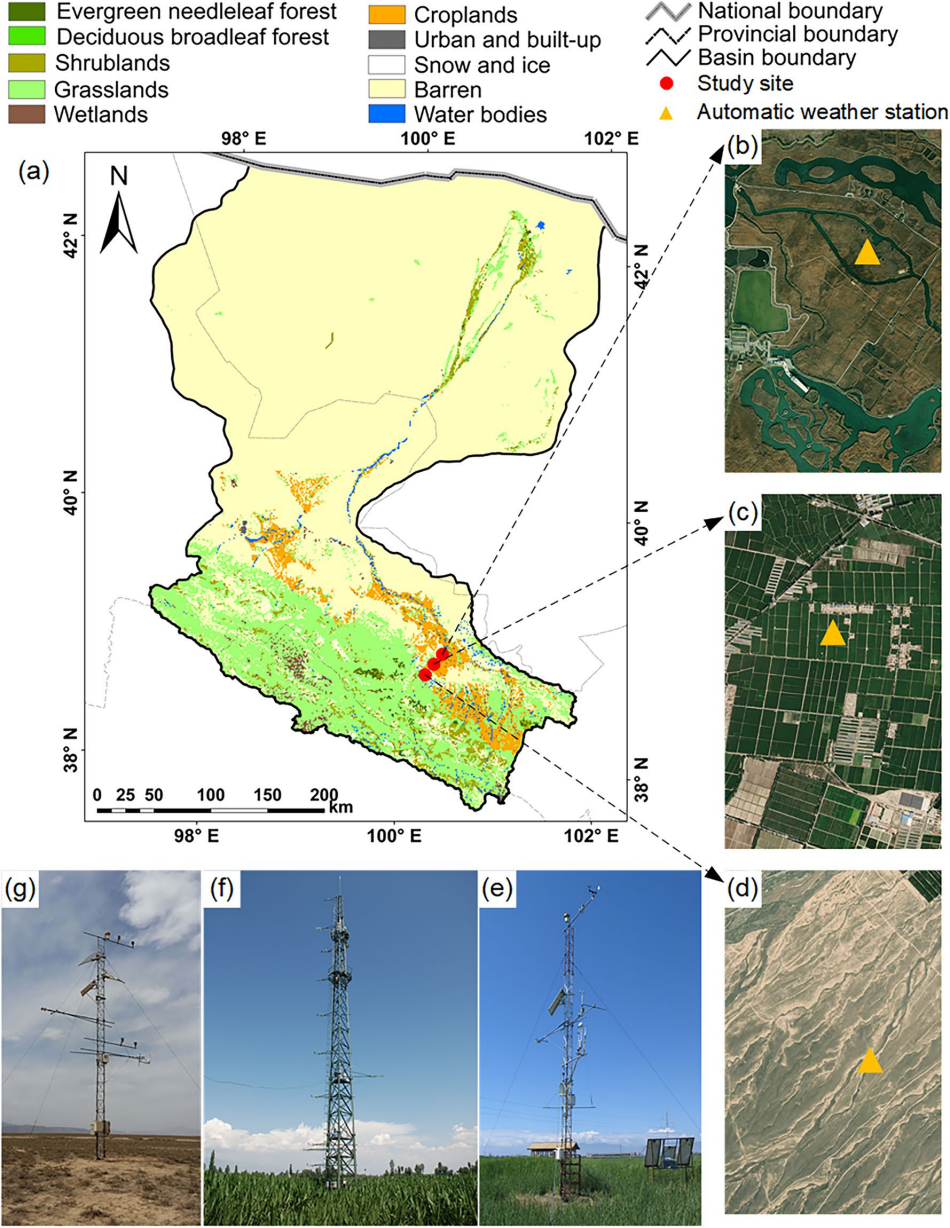
The study sites in the midstream section of the Heihe River Basin. Panel (**a**) illustrates the land cover of the Heihe River Basin. Panels (**b**–**d**) show the three selected representative study areas: the Wetland site, Daman site, and Huazhaizi site, respectively. Panels (**e**–**g**) provide photographs of the automatic weather stations within these three study areas.

This study further refines the midstream region into three representative rectangular experimental areas, aligned with three automatic weather stations from the Heihe Watershed Allied Telemetry Experimental Research (HiWATER) program (Fig. 1). These automatic weather stations are the Daman station (100.372°E, 38.856°N, 1,556 m a.s.l.), the Huazhaizi station (100.320°E, 38.766°N, 1,731 m a.s.l.), and the Wetland station (100.446°E, 38.975°N, 1,460 m a.s.l.). The land cover types surrounding the Daman station primarily consist of farmland, along with orchards, residential areas, shelterbelts, and roads. The Huazhaizi station is located in a desert area sparsely populated by low shrubs such as *Kalidium foliatum*. The Wetland station is surrounded by reed wetlands, as well as waterbodies, residential areas, grasslands, and roads. To avoid confusion, in this study, the term “station” specifically refers to an automatic weather station, while “site” denotes the UAV flight area where the automatic weather station is located. The distinct geographical and ecological conditions of these three areas offer valuable insights for research on arid and semiarid regions.

### Scientific objectives

The MUSOES-UAV experiment focuses on monitoring the land surface thermal states and spectral characteristics in arid and semiarid regions, leveraging ground-based, UAV-based, and satellite observations to achieve multi-scale measurements of representative areas. Its objectives are diverse and interdisciplinary, addressing critical challenges in both quantitative and qualitative remote sensing. The scientific objectives of MUSOES-UAV can be summarized as follows:

1. *Establishing Reproducible UAV Remote Sensing Experiment Procedures and Identifying Data Quality Issues*: Establish comprehensive, highly reproducible UAV remote sensing experiment procedures tailored for arid and semiarid regions using widely accessible commercial platforms. Investigate and identify data quality issues inherent in UAV TIR and multispectral data, and implement robust workflows to enhance data quality.
2. *Supporting Multi-Source Data Geometric Registration and Multi-Scale TIR Data Transformation*: Develop methods for geometric registration of UAV-based multi-source remote sensing data, including multispectral and TIR imagery. Investigate the transformation relationships between TIR data (i.e., BT or LST) across different spatial scales, using UAV platforms as intermediaries. This includes providing a reliable testbed for models linking satellite pixels, UAV observations, and ground measurements, thereby advancing the understanding of spatial scale variations in ecological and environmental factors in these regions.
3. *Providing Input Parameters for Estimating Surface Hydrological and Carbon Fluxes and Exploring Temporal Dynamics*: Provide input parameters for estimating surface hydrological and carbon fluxes, such as soil moisture, sensible heat flux, latent heat flux, and gross primary productivity. Utilize long-term observation datasets spanning the vegetation growing season to examine critical changes in surface and vegetation conditions at key temporal nodes. This dynamic perspective aims to uncover fundamental patterns in arid and semiarid ecosystems.

### Observation system and flight experiments

#### UAV platforms

**Fig. 2:**
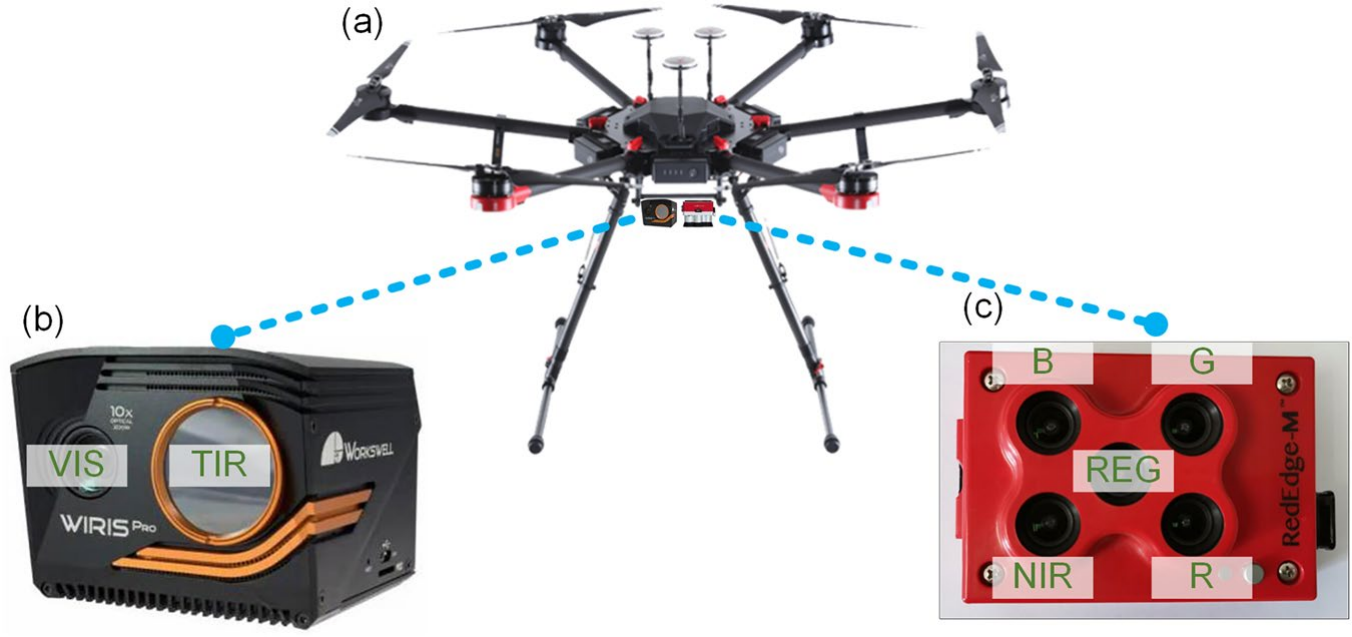
The adopted UAV platform (**a**), the thermal camera (**b**), and the multispectral camera (**c**).

The UAV platform utilized in this experiment is the DJI Matrice 600 Pro hexacopter (Fig. 2a). This multirotor UAV weighs approximately 10 kg (including batteries) and has a recommended maximum takeoff weight of 15.5 kg. It achieves a maximum ascent speed of 5 m/s and a descent speed of 3 m/s, withstanding wind speeds up to 8 m/s, ensuring reliable performance under variable weather conditions during flights. The UAV’s maximum horizontal flight speed reaches 18 m/s in windless conditions, and it supports takeoff altitudes up to 4500 m. Equipped with six fully charged 5700 mAh batteries, the UAV can safely operate for approximately 20 minutes per flight. The platform can be manually controlled via a remote controller operating at 2.4 GHz or programmed for automated flights using an iPad running DJI GS Pro and DJI Go software, enabling real-time monitoring of flight status, battery health, and image acquisition to ensure operational safety.

The Workswell WIRIS Pro, a high-performance scientific-grade dual-sensor thermal camera featuring a TIR lens and a high-definition (HD) visible-light (VIS) lens (Fig. 2b), is mounted on the UAV along with an integrated GPS module (Fig. 2b). The TIR lens operates within a spectral range of 7.5–13.5 μm, with a temperature measurement range of −25 °C to 150 °C, a sensitivity of 30 mK, and a nominal accuracy of ±2 °C or ±2% of the reading, whichever is greater (in °C). The lens provides a field of view (FOV) of approximately 45° × 36° and captures images with a resolution of 640 × 512 pixels, saved in “.tiff” and “.jpg” formats. The HD visible-light lens features autofocus and image stabilization, capturing 1920 × 1080-pixel images in “.jpg” format. The thermal camera is mounted on a factory-supplied three-axis gimbal, allowing flexible orientation; in this study, the camera was consistently oriented downward. The camera settings were standardized across all flights, with emissivity set to 1 to obtain true brightness temperatures while avoiding the complexity of varying land surface emissivity.

Additionally, the UAV platform carries a Micasense RedEdge-M multispectral camera (Fig. 2c), equipped with five lenses corresponding to distinct spectral bands: near-infrared (NIR), red-edge (REG), red (R), green (G), and blue (B). The central wavelengths of these bands are 840 nm (NIR), 717 nm (REG), 668 nm (R), 560 nm (G), and 475 nm (B), with full-width at half-maximum (FWHM) values of 40 nm, 10 nm, 10 nm, 20 nm, and 20 nm, respectively. This specific 5-band multispectral configuration was deliberately selected because it captures the most critical spectral features for vegetation monitoring (including the red-edge and near-infrared plateau) while perfectly aligning with the core broadband configurations of mainstream satellite missions (e.g., Sentinel-2 and Landsat). This alignment is crucial for fulfilling the dataset’s objective of facilitating cross-scale validation and upscaling models, while also maintaining optimal UAV flight endurance for broader spatial coverage.

The lenses have a horizontal FOV of 47.2°, capturing images with a resolution of 1280 × 960 pixels in “.tiff” format. The camera is equipped with a downwelling light sensor and a GPS module, along with a calibration panel for band-specific data correction. Mounted on a custom-built anti-vibration bracket beneath the UAV, the camera captures nadir images. The ample underside space of the DJI Matrice 600 Pro allows the simultaneous installation of the multispectral camera and thermal camera without interference. The parameters for the multispectral camera were also standardized across all flight missions. These instruments and platforms have been widely used in our previous research^20–23,25,26,35^.

### Ground measurements

During the UAV flight missions, the automatic weather stations within the flight areas operated continuously, collecting a range of meteorological variables. At the Daman station, wind speed, wind direction, air temperature, and relative humidity sensors were installed at seven height levels (3 m, 5 m, 10 m, 15 m, 20 m, 30 m, and 40 m). A barometer was mounted at 2 m on the tower, while a four-component radiometer was installed at 12 m. At the Huazhaizi station, the wind speed, wind direction, air temperature, and relative humidity sensors were installed at two height levels (5 m and 10 m). The barometer was housed within a waterproof enclosure, while the four-component radiometer was mounted at 6 m. The Wetland station followed a similar sensor arrangement to the Huazhaizi site, with minor differences: the barometer was installed at 2 m on the tower, and the wind direction sensor was placed only at 10 m. Data from the automatic weather stations at all sites were transmitted in real-time to a data management and control system via wireless transmission modules for subsequent processing. Detailed specifications of the sensors deployed at each site are provided in Table 1. In addition to standard meteorological variables, eddy covariance systems were installed at each station to measure sensible heat flux, latent heat flux, carbon dioxide flux, and methane flux. Notably, a large-aperture scintillometer and an optical-microwave scintillometer were deployed at the Daman station to capture sensible and latent heat fluxes over a broader spatial scale. As flux measurements are not the primary focus of this study, detailed descriptions are omitted here; further information can be found in Liu *et al*.^28,29^. While the long-term continuous observations from these automatic weather stations (maintained by the authors’ team) are part of a broader observational network, we have specifically extracted, cleaned, and temporally aligned the high-frequency ground data corresponding exactly to the UAV flight campaigns. By bundling this strictly synchronized ground-truth data with the UAV imagery, this data descriptor provides a highly cohesive, “ready-to-use” multi-scale dataset, thereby saving future users significant effort in data retrieval and temporal-spatial matching, and facilitating immediate downstream applications such as satellite product validation and scale-transformation studies.

**Table 1:** Information on sensors deployed at each automatic weather stations.

Station	Variables	Sensor	Manufacturer
Daman	Wind speed and direction	WindSonic	Gill, UK
	Air temperature and relative humidity	AV-14TH	Avalon, USA
	Four-component radiation	PSP&PIR	Eppley, USA
Huazhaizi	Wind speed and direction	WindSonic	Gill, UK
	Air temperature and relative humidity	HMP45AC	Vaisala, Finland
	Four-component radiation	CNR1	Kipp&Zonen, the Netherlands
Wetland	Wind speed and direction	WindSonic	Gill, UK
	Air temperature and relative humidity	HMP45AC	Vaisala, Finland
	Four-component radiation	CNR1	Kipp&Zonen, the Netherlands

In addition to the automated observations, Apogee SI-111 TIR radiometers were deployed during each UAV flight mission to capture BT data for specific land cover types. These TIR radiometers were calibrated prior to deployment using a Fluke infrared calibrator. At the Daman site, the observed targets included cornfields, roads, and grasslands. At the Huazhaizi site, the target was bare soil, while at the Wetland site, observations focused on reed wetlands, roads, and grasslands. Furthermore, at each site, the sky BT at a zenith angle of 53° was also recorded^36^. All TIR radiometers were connected to Campbell CR300 or CR800 series data loggers, enabling real-time data recording and storage.

### UAV survey flights

The UAV flight areas were designed based on the coverage of MODIS pixels and the footprint of flux measurement instruments at each station to support potential satellite-synchronized observations and flux-related studies. Specifically, each site required coverage of two MODIS pixels while ensuring that the observational footprints of the eddy covariance systems or large-aperture scintillometers were fully included. To balance mission efficiency and battery endurance, the UAV flight altitude was set to 300 m above ground level, resulting in a spatial resolution of approximately 0.4 m for TIR images and 0.2 m for multispectral images.

Given the relatively weaker texture details in TIR images compared to visible light images^37^, the overlap rates for TIR images were set to high values to improve the success rate of subsequent image mosaicking (lateral overlap >70%, longitudinal overlap >80%). Flight path planning was performed using the DJI GS Pro software, which generated a Z-shaped grid flight route based on the flight altitude and sensor FOV. Due to the large coverage area, multiple battery sets were required to complete each mission. The UAV was programmed to return to the takeoff point at a designated waypoint for battery replacement, after which it resumed the remaining observations from the same waypoint.

The specific flight mission details for each site are provided in Table 2. A total of 15 flight missions were conducted from June to October 2020. To minimize the influence of environmental factors^38^, flight missions were performed on days with relatively stable weather conditions and low wind speeds. Prior to each flight, the multispectral camera was manually operated to capture images of a ground calibration panel to enable reflectance correction during post-processing. The thermal camera was configured to capture images automatically at fixed time intervals (2 seconds), while the multispectral camera began image acquisition at the preset altitude, also at 2-second intervals.

**Table 2:** Flight mission details for each site.

Site	Growing season stage	Flight date (yyyymmdd)	Duration (hh:mm)	Strip count	Image count (TIR/Multispectral)
Daman	Early	20200618	10:56–13:32	21	NA/9625
	Middle	20200713	10:03–12:25	21	2010/7990
	Middle	20200820	10:46–13:04	21	3821/7520
	Late	20200919	10:33–12:52	21	3832/4810
	Harvest	20201019	10:48–13:27	21	NA/6180
Huazhaizi	Early	20200614	11:01–14:54	19	NA/8845
	Middle	20200714	15:52–17:10	19	1083/5895
	Middle	20200820	16:51–18:37	19	5935/5695
	Late	20200919	14:58–16:28	19	1328/4410
	Harvest	20201020	11:08–12:47	19	2990/4905
Wetland	Early	20200618	11:22–13:10	16	NA/6890
	Middle	20200714	10:43–12:37	16	1720/5530
	Middle	20200821	15:17–16:42	16	2860/4960
	Late	20200920	10:41–12:09	16	1182/4230
	Harvest	20201021	11:16–12:34	16	2409/4075

Multispectral data were successfully collected for all flight missions. However, due to technical issues and maintenance of the thermal camera, TIR data were not acquired in June 2020. In October 2020, TIR data were not successfully collected for Daman site.

### Data processing and quality control

#### UAV thermal infrared data

The thermal camera used in this study is an uncooled thermal imager, where the temperature of the detector elements is not actively cooled but instead varies according to the ambient temperature and the thermal characteristics of the sensor itself^39–41^. Particularly during large-scale flight missions, environmental factors such as wind speed, air temperature, and sunlight can significantly affect the thermal sensor, leading to substantial temperature drift. This means that the temperature readings captured by the sensor can deviate significantly from the true temperature of the target, and these changes are not predictable in advance. Although the thermal imager is equipped with a built-in temperature calibration function, practical experience has shown that this calibration is insufficient to fully compensate for the effects of temperature drift^42–44^. To mitigate the impact of temperature drift, we proposed a method based on the fitting of the digital number (DN) probability density function and radiative transfer simulation, referred to as the DRAT method^35^. This method primarily posits that the temperature drift present in TIR images consists of a series of biases, with different images typically corresponding to different biases.

Initially, the representative digital number (RDN) value is identified from the probability density function of the DN values in the images. This RDN characterizes the average BT of the dominant land cover type (i.e., the land cover type with the largest area proportion) within the image. Subsequently, a reference TIR image is selected, and the difference between the RDNs of the other images and the RDN of the reference image is calculated. This is followed by the correction of the original image sequence through bias correction, which ensures a normalized TIR image sequence with the temperature drift removed. The process can be expressed by Eq. (1).

1 $$\{\begin{array}{c}\Delta {\mu }_{i}={\mu }_{i}-{\mu }_{1},\,i\ge 2\\ {{\boldsymbol{\mu }}}_{{\rm{norm}},i}={{\boldsymbol{\mu }}}_{i}-\Delta \mu ,\,i\ge 2\end{array}$$

where *μ*_*i*_ is the RDN of the *i*-th image; *μ*_1_ is the RDN of the reference; Δ*μ*_*i*_ represents the RDN difference of the *i*-th frame relative to the reference image; **μ**_*i*_ is the original DNs of the *i*-th image; **μ**_norm,*i*_ is the normalized DNs of the *i*-th image.

Given that the difference between RDNs is actually a coupling of the temporal effect and temperature drift (Eq. 2), this method also roughly achieves temporal normalization of temperatures through bias correction.

2 $${\mu }_{i}={\psi }_{i}+{\nu }_{i},\,i\ge 2$$

where *ψ*_*i*_ denotes the temperature drift of the *i*-th image relative to the reference image; *ν*_*i*_ represents the temporal effect of the *i*-th image relative to the reference image.

However, this introduces a certain limitation as it inherently assumes the temporal effect (*v*_*i*_) is consistent across the flight for all land cover types. Because different land covers exhibit varying temperature change rates, residual temporal differences may not be fully eliminated for non-dominant land cover types. Nevertheless, despite this limitation, the corrected BT mosaics still demonstrate significant improvements in image quality and quantitative accuracy (as detailed in the Technical Validation section).

The normalized TIR image sequences were processed using Pix4D mapper software to generate TIR orthomosaic maps. Pix4D mapper is widely utilized in industrial and academic photogrammetry applications due to its high level of automation, robust data processing capabilities, and efficient hardware utilization^45^. In this study, the “Thermal Camera” template with default parameters was used for the mosaicking process. The core algorithmic steps in Pix4D mapper for generating TIR orthomosaics include the following: First, feature points are detected and extracted from the TIR images. Next, the camera’s internal and external parameters are optimized using the bundle adjustment method to enhance geometric consistency between images. Based on this, sparse point clouds are generated through feature point matching, establishing preliminary 3D spatial relationships between images. Subsequently, dense point clouds are produced using multi-view stereo matching algorithms, which provide a foundation for further geometric correction. Orthorectification is then applied, projecting images from oblique views onto a ground plane to eliminate geometric distortions and produce orthorectified maps. Finally, the processed orthomosaic maps are exported in standard GeoTIFF format, including georeferencing information and high-quality imagery, facilitating subsequent analysis and applications. All images are aligned to the WGS84 Universal Transverse Mercator (UTM) Zone 47 N coordinate system.

To ensure consistency across datasets from different months, the TIR orthomosaic maps from July 2020 for the three sites were used as reference images. Orthomosaic maps from other months were manually registered to these reference maps using ArcMap software. Additionally, to address variations in spatial resolution, all images were resampled to a uniform spatial resolution of 0.4 m. After registration and resampling, the TIR orthomosaics were converted to BT orthomosaics using standard conversion coefficients provided by the thermal camera manufacturer, as shown in Eq. (3).

3 $$BT=DN\cdot 0.025-100$$

where *BT* is brightness temperature in °C.

Since the temperature normalization process during temperature drift removal aligns the TIR image sequence to the temperature level of a reference image, some residual temperature drift may still be present. To further refine the BT orthomosaics, calibration was performed using data from ground-based SI-111 TIR radiometers. As a result, we obtained reliable, geometrically consistent, and spatially uniform BT products suitable for advanced analysis.

### UAV multispectral data

The multispectral images were also processed using Pix4D mapper software for automated mosaicking. The software is pre-configured with the lens parameters of the RedEdge-M multispectral camera, making the processing workflow more efficient. Upon importing the multispectral images from all five bands into the software, Pix4D mapper automatically recognizes and groups the images by their respective spectral bands. The software then calculates the radiance values from the original DN values of the multispectral images using Eq. (4) (https://support.micasense.com/hc/en-us/articles/115000351194-Radiometric-Calibration-Model-for-MicaSense-Sensors, last access: March 3, 2025).

4 $${L}_{i}=V(x,y)\cdot \frac{{a}_{1}}{g}\cdot \frac{p(x,y)-{p}_{{\rm{BL}}}}{{t}_{{\rm{e}}}+{a}_{2}y-{a}_{3}{t}_{{\rm{e}}}y}$$

where *x* and *y* represent the column and row indices of the pixel, respectively; *V*(*x*,*y*) is the lens vignetting correction function at pixel location (*x*,*y*); *a*_1_, *a*_2_, and *a*_3_ are the radiometric calibration coefficients; *g* is the sensor gain; *p*(*x*,*y*) denotes the normalized raw pixel value at location (*x*,*y*); *p*_BL_ is the normalized black level value; *t*_e_ is the exposure time of the imager; *L*_*i*_ is the radiance of band *i* (*i = *1,2,3,4,5) in W·m^−2^·sr^−1^·nm^−1^.

The RedEdge-M multispectral camera employs a radial vignetting model to correct for the sensitivity reduction observed in pixels located farther from the image center, as described in Eq. (5).

5 $$\{\begin{array}{c}r=\sqrt{{(x-{c}_{{\rm{x}}})}^{2}+{(y-{c}_{{\rm{y}}})}^{2}}\\ k=1+{k}_{0}\cdot r+{k}_{1}\cdot {r}^{2}+{k}_{2}\cdot {r}^{3}+{k}_{3}\cdot {r}^{4}+{k}_{4}\cdot {r}^{5}+\\ {I}_{{\rm{c}}}(x,y)=\frac{I(x,y)}{k}=V(x,y)\cdot I(x,y)\end{array}{k}_{5}\cdot {r}^{6}$$

where *c*_x_ and *c*_y_ are the coordinates of the vignetting center; *k*_0_, *k*_1_, *k*_2_, *k*_3_, *k*_4_, and *k*_5_ are coefficients stored in the image metadata; *r* represents the distance, in pixels, between the pixel at (*x*,*y*) and the vignetting center; *k* is the correction factor, and *V*(*x*,*y*) is its reciprocal; *I*(*x*,*y*) denotes the original radiance at pixel location (*x*,*y*), while *I*_c_(*x*,*y*) is the corrected radiance at the same location.

Then, the radiance values for each band were converted to reflectance using Eq. (6), based on the calibration panel images captured prior to each flight segment.

6 $$\{\begin{array}{c}{F}_{i}=\frac{{\rho }_{i}}{avg({L}_{i})}\\ {\alpha }_{i}={F}_{i}\cdot {L}_{i}\end{array}$$

where *ρ*_*i*_ represents the reflectance of the calibration panel in the *i*-th band; *avg*(*L*_*i*_) denotes the average radiance of all pixels covered by the calibration panel in the *i*-th band; *α*_*i*_ is the reflectance in the *i*-th band.

Due to battery replacements and instrument adjustments, a complete UAV flight mission for a given site is divided into multiple segments. This means that prior to each segment, the multispectral camera captures images of a ground calibration panel for subsequent correction. As a result, the stitching of multispectral images is performed on a per-segment basis rather than combining all images from the entire flight mission at once. Under this approach, image data from a single segment produce five sub-orthomosaics corresponding to different spectral bands. If a flight mission is divided into *N* segments, a total of 5 *N* sub-orthomosaics will be generated. The stitching of multispectral images was conducted using the “Ag Multispectral” template in Pix4D mapper with default parameters, and radiometric correction was performed using the “Camera and Sun Irradiance” option. The core algorithm steps for generating multispectral orthomosaics in Pix4D mapper are similar to those for TIR orthomosaics. However, instead of producing thermal radiance maps, the final output for multispectral data consists of reflectance maps for each spectral band.

In practice, systematic differences in reflectance may exist among different sub-orthomosaics^46^. This means that the reflectance of the same area can exhibit significant inconsistencies across sub-orthomosaics. If all sub-orthomosaics from a single flight mission are directly stitched together, the resulting composite reflectance orthomosaic may display noticeable seams and variations in brightness, leading to a substantial degradation in image quality. To address this issue, we employed a radiometric relative normalization method based on linear regression to correct the radiometric inconsistencies between sub-orthomosaics. First, a reference sub-orthomosaic (e.g., from the first flight segment) was selected. Using the Scale-Invariant Feature Transform (SIFT) algorithm^47^, corresponding tie points in the overlapping regions of adjacent sub-orthomosaics (e.g., the second flight segment) were identified. The linear regression coefficients for reflectance values at corresponding locations were then estimated using the least squares regression. These regression coefficients were applied to normalize the reflectance values of the second flight segment’s sub-orthomosaic, achieving radiometric relative normalization as expressed in Eq. (7).

7 $$\{\begin{array}{c}({a}_{1},{b}_{1})=LSR({\bf{R}}{\bf{E}}{{\bf{F}}}_{{\bf{1}},{\bf{t}}{\bf{i}}{\bf{e}}},{\bf{R}}{\bf{E}}{{\bf{F}}}_{{\bf{2}},{\bf{t}}{\bf{i}}{\bf{e}}})\\ {\bf{R}}{\bf{E}}{{\bf{F}}}_{{\bf{2}},{\bf{n}}{\bf{o}}{\bf{r}}{\bf{m}}}={a}_{1}\cdot {\bf{R}}{\bf{E}}{{\bf{F}}}_{{\bf{2}}}+{b}_{1}\end{array}$$

where **REF**_**1,tie**_ represents the collection of reflectance values at the tie points on the reference sub-orthomosaic (i.e., from the first flight segment); **REF**_**2,tie**_ represents the collection of reflectance values at the corresponding tie points on the original adjacent sub-orthomosaic (i.e., from the second flight segment); *LSR*() denotes the least squares regression operator; *a*_1_ and *b*_1_ are linear regression coefficients between the reference and the original adjacent sub-orthomosiacs; **REF**_**2**_ represents the original adjacent sub-orthomosaic; **REF**_**2,norm**_ represents the radiometrically normalized adjacent sub-orthomosaic.

Subsequently, the remaining sub-orthomosaics can be sequentially radiometrically normalized, bringing their reflectance values to the level of the reference sub-orthomosaic, as shown in Eq. (8).

8 $$\{\begin{array}{c}({a}_{j},{b}_{j})=LSR({\bf{R}}{\bf{E}}{{\bf{F}}}_{{\bf{j}},{\bf{n}}{\bf{o}}{\bf{r}}{\bf{m}},{\bf{t}}{\bf{i}}{\bf{e}}},{\bf{R}}{\bf{E}}{{\bf{F}}}_{{\bf{j}}{\boldsymbol{+}}1,{\bf{t}}{\bf{i}}{\bf{e}}})\\ {\bf{R}}{\bf{E}}{{\bf{F}}}_{{\bf{j}}{\boldsymbol{+}}1,{\bf{n}}{\bf{o}}{\bf{r}}{\bf{m}}}={a}_{j}\cdot {\bf{R}}{\bf{E}}{{\bf{F}}}_{{\bf{j}}{\boldsymbol{+}}1}+{b}_{j}\end{array},2\le j\le N-1$$

where **REF**_**j,norm,tie**_ represents the collection of reflectance values at the tie points on the radiometrically normalized *j*-th sub-orthomosaic; **REF**_**j+1,tie**_ represents the collection of reflectance values at the corresponding tie points on the (*j* + 1)-th original sub-orthomosaic; *a*_j_ and *b*_j_ are linear regression coefficients between the *j*-th radiometrically normalized and the (*j* + 1)-th original sub-orthomosiacs; **REF**_**j+1**_ represents the original (*j* + 1)-th sub-orthomosaic; **REF**_**j+1,norm**_ represents the radiometrically normalized (*j* + 1)-th sub-orthomosaic; *N* is the count of flight segments.

Finally, the radiometrically normalized sub-orthomosaics are stitched and stacked to generate a complete multispectral mosaic covering the entire flight area, which includes five spectral bands. These images are exported in the standard GeoTIFF format with the coordinate system set to WGS84 UTM Zone 47 N. Similarly, to eliminate inconsistencies in geometry and spatial resolution between different monthly datasets, the orthomosaics from July 2020 across the three sites are used as a reference. The orthomosaics from other months are manually georeferenced to the reference mosaics using ArcMap software, and the spatial resolution of all mosaics is resampled to 0.2 m. Additionally, atmospheric correction of reflectance across various spectral bands was conducted utilizing the Second Simulation of the Satellite Signal in the Solar Spectrum (6S) model, employing a mid-latitude summer atmospheric profile for the simulation.

### Ground measurements

The observational instruments of the automatic weather stations were calibrated and underwent consistency checks prior to installation. Regular on-site inspections and maintenance were conducted monthly to ensure the reliability of the equipment. The observational data from the automatic weather stations were processed using the Heihe Watershed Internet of Things (IoT) Observation System. This robust system supports remote reception and storage of field data, real-time data visualization and interaction, online monitoring of sensor status, and anomaly alerts. Meteorological data processing followed an automated workflow based on the following principles: (1) all observational data were averaged at 10-minute intervals, resulting in 144 data entries per day, with missing data filled using the placeholder value −6999; (2) observations with values deemed physically implausible were excluded and also filled with −6999. Furthermore, prior to data archiving, quality assurance measures such as self-checks, cross-validation, and expert reviews were conducted to ensure data quality. A brief overview of the meteorological data processing is provided here; further details can be found in Liu *et al*.^28,29^.

For data collected by the ground-deployed SI-111 TIR radiometers, the sampling interval was set to 5 seconds. At the end of each flight mission, the observational data were exported and stored on hard drives. BT data were calibrated using coefficients derived from dedicated calibration experiments, and all observational data were organized into standard “.xlsx” format files.

## Data Records

The datasets are freely available for download from the National Tibetan Plateau Center: 10.11888/Terre.tpdc.302412^48^. The datasets include BT orthomosaics corrected for temperature drift, multispectral orthomosaics corrected with relative radiometric normalization, and NDVI maps derived from these orthomosaics. Also included are observations from automatic weather stations and ground-based thermal infrared (TIR) radiometers. The spatial resolution of the BT orthomosaics is 0.4 m, while the multispectral orthomosaics and NDVI maps have a spatial resolution of 0.2 m. All datasets have been relatively georeferenced using data from July 2020 as the baseline and are provided in GeoTIFF format. The observational data from the automatic weather stations and ground-based infrared radiometers are organized in.xlsx format. All temporal references for the datasets are in China Standard Time.

For clarity and ease of use, the datasets are stored in a hierarchical folder structure following the “Product Type/Site/Time” format. The “Product Type” folders include BT (for brightness temperature orthophotos), Mul (for multispectral orthophotos), NDVI (for NDVI maps), FT (for automatic weather station data), and TIR (for ground-based infrared radiometer data). The “Site” subfolders correspond to the data collection locations: Daman, Wetland, and Huazhaizi. Individual files are named using the YYYYMMDD_XX.tif/.xlsx convention, where YYYY, MM, and DD represent the year, month, and day, and XX is a data description. For example, the file NDVI/Daman/20200618_NDVI.tif represents the NDVI map for Daman Station on June 18, 2020. For time-series data, the naming convention is YYYYMMDD_to_YYYYMMDD_Site.xlsx, as exemplified by Flux_Tower/20200601_to_20201031_Daman.xlsx, which contains automatic weather station data for Daman Station from June 1, 2020, to October 31, 2020.

To facilitate the interpretation and utilization of the ground-based measurements, a data dictionary explicitly listing the core variables, their descriptions, and units for the automatic weather stations and ground TIR radiometers is provided in Table 3. Please note that while the raw weather station files may contain additional parameters, this table focuses on the primary meteorological and radiometric variables relevant to this data descriptor.

**Table 3:** Data dictionary for the key variables in the ground-based measurement files.

Variable	Unit	Explanation
Timestamp	YYYY/M/D H:MM:SS	Date and time of data recording (local time).
Ta_level_Avg	°C	Average air temperature. (The “level” suffix denotes the observation level, with smaller numbers indicating lower heights above the ground).
RH_level_Avg	%	Average relative humidity.
WS_level_Avg	m/s	Average wind speed.
WD_level_Avg	degree	Average wind direction.
DR_Avg	W/m^2^	Average downwelling shortwave radiation.
UR_Avg	W/m^2^	Average upwelling shortwave radiation.
DLR_Cor_Avg	W/m^2^	Average downwelling longwave radiation.
ULR_Cor_Avg	W/m^2^	Average upwelling longwave radiation.
Rn_Avg	W/m^2^	Average net radiation.
IRT_level_Avg	°C	Average BT observed by the automatic weather station’s TIR radiometer.
Press_Avg	hPa	Average atmospheric pressure.
Type_Temp	K	BT observed by the ground-based TIR radiometer. (The “Type” prefix denotes the specific surface target being observed).

## Technical Validation

### BT mosaics

**Fig. 3:**
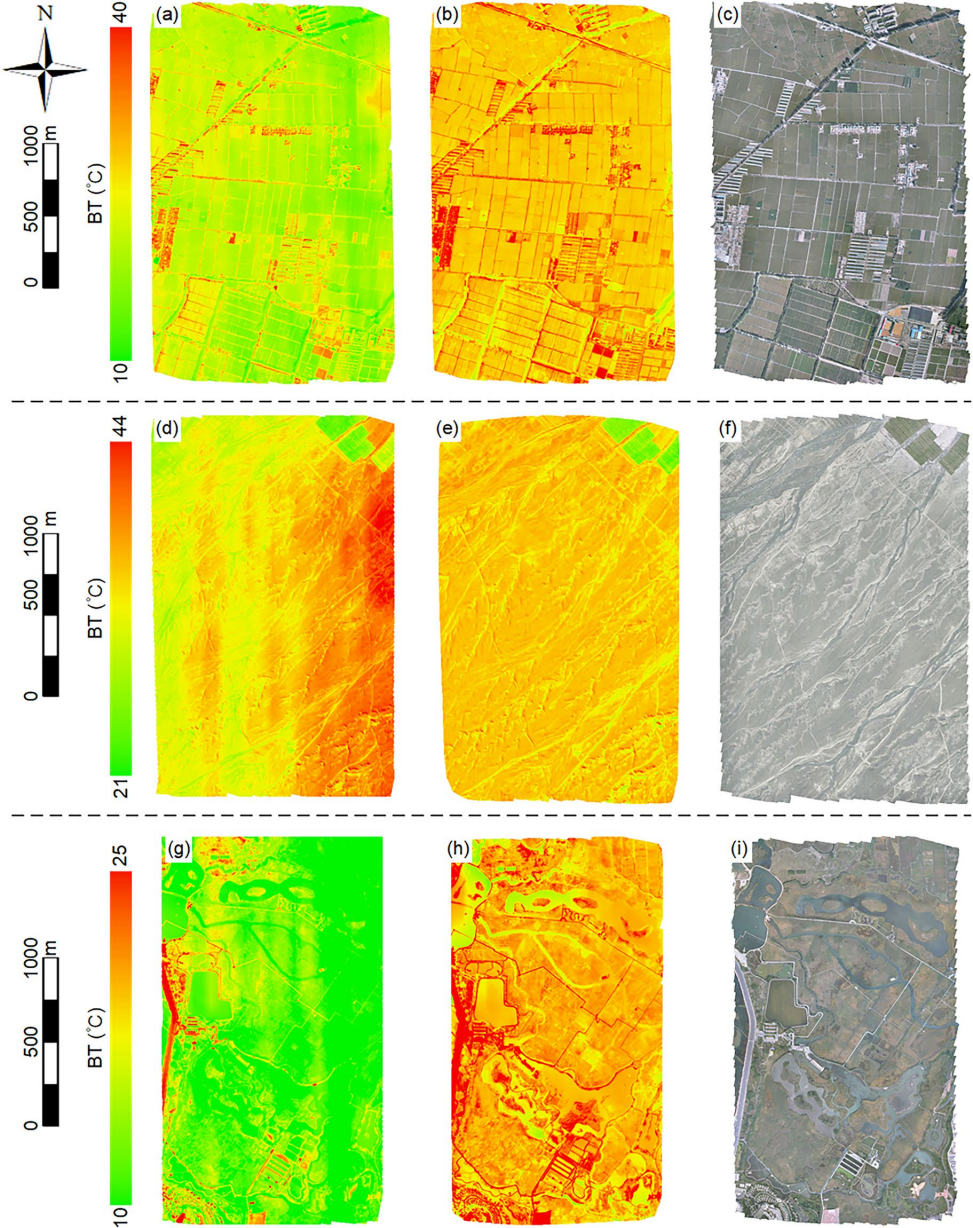
Examples of BT and VIS orthomosaics of the three sites in Semptember 2020. The first column represents BT orthomosaics before temperature drift removal, the second column shows BT orthomosaics after temperature drift removal, and the third column displays VIS orthomosaics as references. The first row corresponds to flight data from the Daman site on September 19, 2020, the second row to the Huazhaizi site on September 19, 2020, and the third row to the Wetland site on September 20, 2020.

All monthly BT data were corrected for temperature drift to ensure the quality of the BT orthomosaics. To illustrate the differences in BT orthomosaic quality before and after temperature drift removal, we use data from the three sites in September 2020 as examples. The BT orthomosaics generated from raw data (Fig. 3a,d,g) and those corrected for temperature drift (Fig. 3b,e,h) are presented. As a reference, high-resolution VIS orthomosaics generated from VIS images acquired during the same flight missions are also provided (Fig. 3c,f,i). Overall, BT orthomosaics derived from raw data exhibit notable anomalies, such as striping patterns and bright or dark streaks, as well as inconsistent relative temperature distributions. In contrast, BT orthomosaics corrected for temperature drift display more reasonable temperature distributions, smoother transitions, and no significant striping anomalies. Moreover, their temperature patterns align well with the land cover types observed in the VIS orthomosaics.

At the site-specific level, the Daman site is primarily covered by farmland, with additional land cover types including roads and buildings. In the raw BT orthomosaic, farmland exhibits significant inter-class temperature differences, with greater variation along the horizontal axis than the vertical axis. A diffuse bright patch is particularly evident in the upper-right corner. Furthermore, the temperature distribution of roads and buildings in the raw BT orthomosaic appears discontinuous, failing to capture the continuity of these features, and in some cases, building temperatures are unrealistically lower than those of farmland. After temperature drift correction, the farmland temperature distribution becomes consistent and uniform, aligning well with the VIS orthomosaic, and the discontinuities in the temperature distribution of roads and buildings are significantly reduced. The Huazhaizi site is predominantly desert, with a small portion of farmland in the upper-right corner. In the raw BT orthomosaic, striping effects are prominent, with irregularly alternating high- and low-temperature regions along the horizontal axis, and substantial inter-class temperature differences. Following temperature drift correction, the uniformity of the BT orthomosaic improves significantly, striping effects disappear, and road features within the desert become clearly identifiable, corresponding well with the VIS orthomosaic. The Wetland site features diverse land cover types, including reed wetlands, water bodies, and other land cover types. The raw BT orthomosaic shows distinct striping effects and anomalous patches, particularly within the reed wetlands and waterbodies, where temperature distribution patterns are chaotic, making it difficult to delineate their boundaries. Additionally, high-temperature features such as roads and buildings occasionally exhibit anomalously low temperatures compared to low-temperature features like waterbodies. After temperature drift correction, the temperature distribution among different land cover types becomes more reasonable, allowing for clear differentiation of surface features, and anomalous relative temperature differences are corrected.

To further evaluate the corrected BT orthomosaics, independent observations from ground-based SI-111 TIR radiometers were used for quantitative validation. The results indicate that the corrected BT data exhibit high accuracy, with a coefficient of determination (*R*²) of 0.97, a mean bias error (MBE) of 0.19 °C, and a root mean square error (RMSE) of 1.51 °C, as detailed in Wang *et al*.^35^. These findings demonstrate that the corrected BT orthomosaics achieve superior performance in both qualitative image quality and quantitative accuracy, thereby supporting both qualitative and quantitative remote sensing studies.

### Multispectral mosaics

**Fig. 4:**
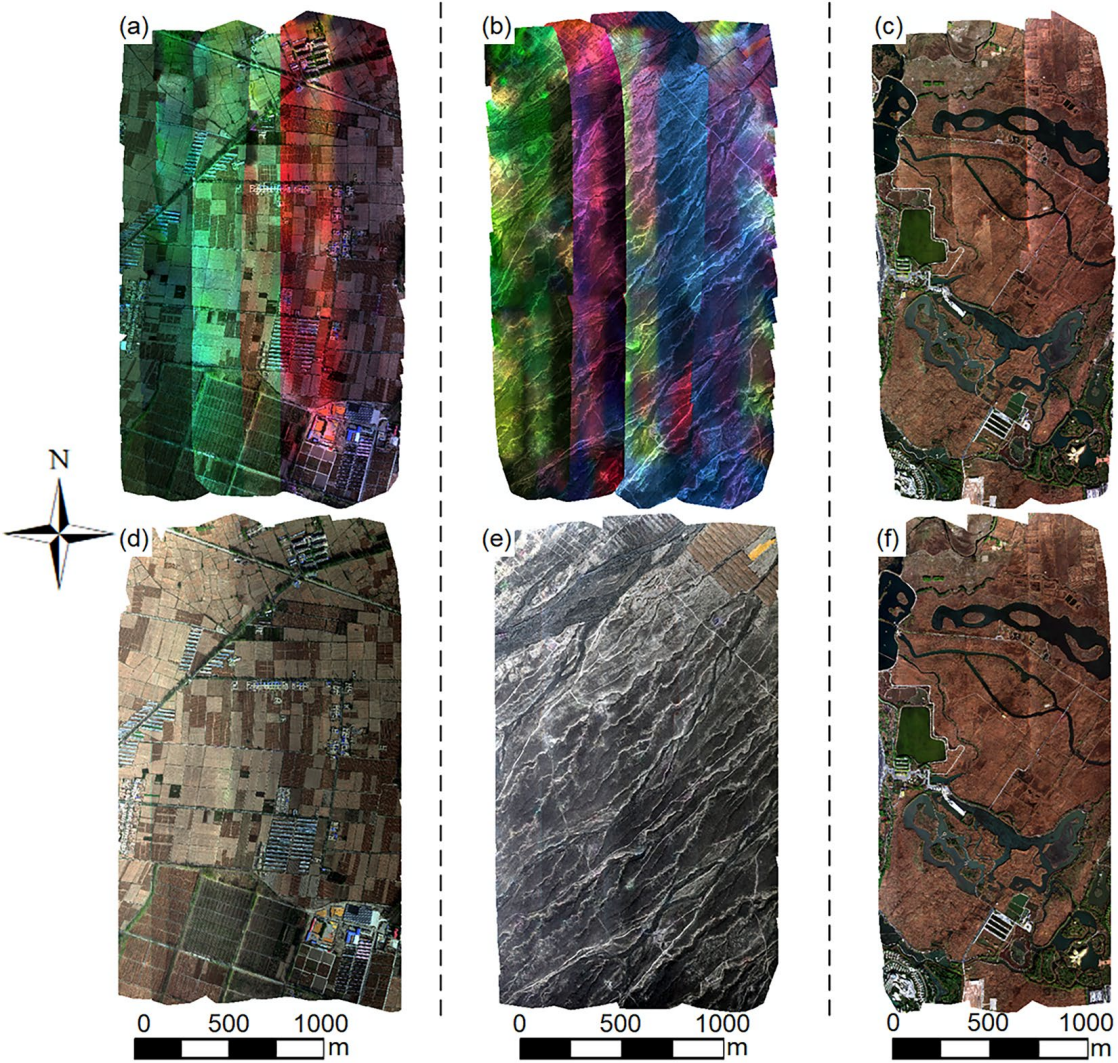
Examples of multispectral orthomosaics for the three sites in October 2020 (composed of R/G/B bands). The first row shows the multispectral orthomosaics before radiometric normalization, and the second row presents the orthomosaics after radiometric normalization. The first column corresponds to data from the Daman site on October 19, 2020, the second column corresponds to data from the Huazhaizi site on October 20, 2020, and the third column corresponds to data from the Wetland site on October 21, 2020.

All multispectral data were subjected to radiometric relative normalization to achieve visually consistent and high-quality images. To compare the effectiveness of two approaches: (1) directly mosaicking sub-orthomosaics from each flight segment without radiometric relative normalization to produce a multispectral orthomosaic covering the entire flight area; and (2) performing radiometric relative normalization on sub-orthomosaics with the first segment as the reference before mosaicking, we analyzed data collected from the three sites in October 2020. Figure 4 illustrates the multispectral orthomosaics generated using the two approaches, with R/G/B bands combined for visualization.

Without radiometric relative normalization, the images exhibited significant anomalies, including evident seam lines and pronounced color inconsistencies (Fig. 4a–c). These issues not only compromised the visual quality of the mosaics but also introduced challenges in subsequent analyses, such as inaccurate vegetation indices or misclassification of land cover types. At the Wetland site, although the color inconsistency was less severe compared to the Daman and Huazhaizi sites, the presence of visible seam lines still undermined the overall integrity of the imagery.

In contrast, the radiometric relative normalization method substantially improved the image quality (Fig. 4d–f). The normalized images demonstrated remarkable color uniformity that closely aligned with the actual ground conditions, with no noticeable striping or pronounced tonal anomalies. Additionally, the suppression of seam lines resulted in smoother transitions between segments, creating a more cohesive and natural-looking mosaic. This improvement is particularly critical for applications requiring precise spatial analysis, such as vegetation mapping, hydrological studies, and land use classification.

Moreover, the normalized images not only enhanced visual consistency but also increased the reliability of quantitative analyses. The method enabled more accurate identification of land cover types, improved classification accuracy, and facilitated temporal comparisons across datasets. Although minor seam lines were still observable in some areas, their impact was negligible compared to the significant enhancements achieved. These results underscore the importance of radiometric relative normalization in ensuring both the visual and analytical quality of multispectral orthomosaics, thereby supporting qualitative and quantitative remote sensing research.

Although the radiometric relative normalization substantially improves the overall mosaic quality, minor residual angular signatures (e.g., Bidirectional Reflection Distribution Function (BRDF) effects) inherent to multi-angle UAV observations may still be visible in the composite imagery (Fig. 4). Because the study area is relatively flat, topographical angular effects are minimal; however, fully correcting for sensor viewing angle effects involves complex BRDF modeling^49,50^. Providing precisely angular-corrected reflectance falls outside the scope of this baseline data descriptor. Instead, we retain these natural radiometric characteristics to transparently present the baseline data quality, which we hope will inspire users to develop and apply targeted angular correction models for their specific downstream analyses.

**Fig. 5:**
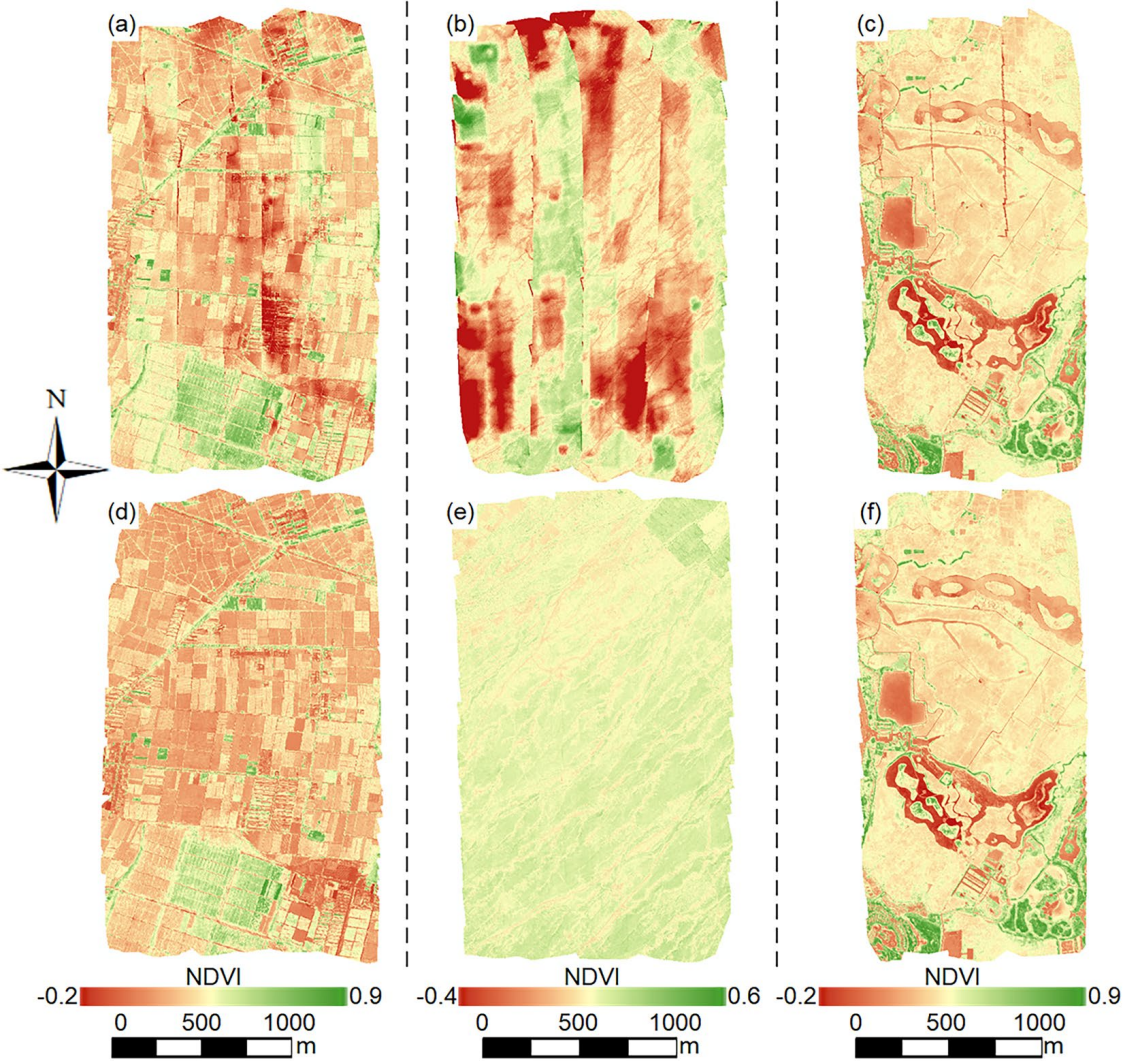
NDVI maps for the three sites in October 2020. The first row displays the NDVI maps derived from multispectral data without radiometric normalization, while the second row shows the NDVI maps derived from multispectral data after radiometric normalization. The first column corresponds to data from the Daman site on October 19, 2020, the second column corresponds to data from the Huazhaizi site on October 20, 2020, and the third column corresponds to data from the Wetland site on October 21, 2020.

To further demonstrate the effectiveness of the multispectral orthomosaics processed using the proposed radiometric relative normalization method in calculating vegetation indices, we used the multispectral data from the three sites (Daman, Huazhaizi, and Wetland) in October 2020 as examples to compute the NDVI. The results before and after radiometric relative normalization are presented in Fig. 5, with the NDVI calculated using Eq. (9).

9 $$NDVI=\frac{re{f}_{{\rm{NIR}}}-re{f}_{{\rm{R}}}}{re{f}_{{\rm{NIR}}}+re{f}_{{\rm{R}}}}$$

where *ref*_NIR_ is the surface reflectance in the near-infrared band, and *ref*_R_ is the surface reflectance in the red band.

As shown in Fig. 5(a–c), the band-based computation partially mitigated the unevenness caused by the lack of radiometric relative normalization, reducing some of the visual artifacts. However, these NDVI images still exhibited significant anomalies, including regions with abnormally high or low values that deviated substantially from the actual ground conditions. Such anomalies introduce considerable uncertainty, limiting the reliability of the data for practical applications.

In contrast, the NDVI images generated from radiometrically normalized multispectral data exhibited significant visual and analytical improvements (Fig. 5d–f). These images displayed smooth transitions with no detectable anomalous regions or seam lines, producing a uniform appearance across all three sites. For example, in late October, the Daman site had low NDVI values overall due to the post-harvest state of cornfields, with only a few areas showing slightly higher NDVI values. The Huazhaizi site, characterized by desert terrain and sparse vegetation, exhibited the lowest NDVI values among the three sites, with almost no regions of high NDVI. Meanwhile, the Wetland site, where reeds had mostly withered by this time, displayed higher NDVI values in certain grassy areas or regions with evergreen trees.

These results clearly demonstrate that NDVI images derived from radiometrically normalized multispectral data are of higher quality and accurately reflect the growth and distribution of surface vegetation. This enhancement ensures the reliability of vegetation indices for supporting relevant research and analysis, particularly in ecological monitoring and land cover studies.

### Ground-based parameters

**Fig. 6:**
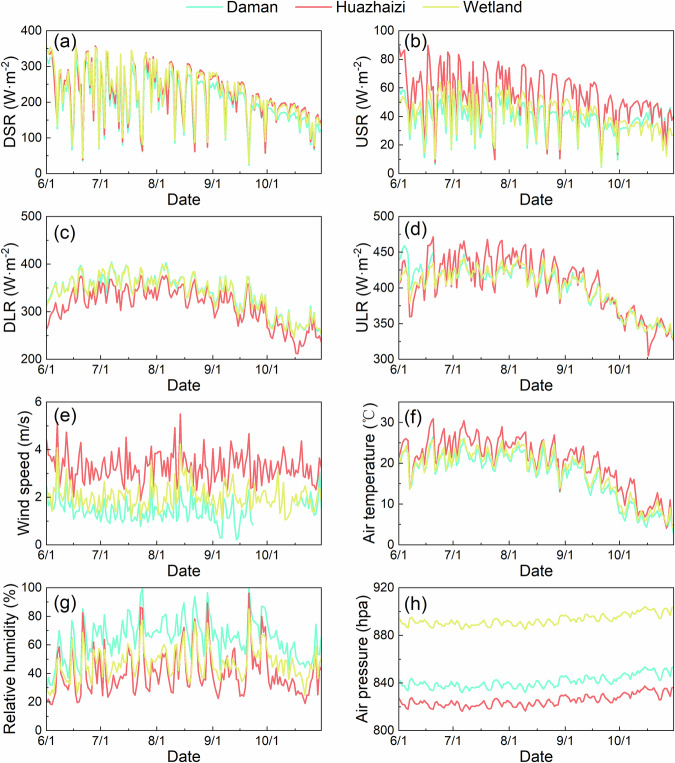
Variations in the daily mean values of key observational parameters recorded by automatic weather stations from June to October 2020. The parameters include downwelling shortwave radiation (**a**), upwelling shortwave radiation (**b**), downwelling longwave radiation (**c**), upwelling longwave radiation (**d**), wind speed (**e**), air temperature (**f**), relative humidity (**g**), and air pressure (**h**). Note that no valid wind speed data were recorded at the Daman site between September 24 and October 17.

Understanding the temporal variations in surface radiation and microclimate is crucial for studying land surface energy balance and hydrothermal exchange processes in arid and semiarid regions. Observations from automatic weather stations provide a valuable foundation for such research. Figure 6 illustrates the daily mean values of representative parameters recorded at the three research sites from June to October 2020. These parameters include downwelling shortwave radiation (DSR), upwelling shortwave radiation (USR), downwelling longwave radiation (DLR), upwelling longwave radiation (ULR), wind speed, air temperature, relative humidity, and air pressure. Notably, wind speed, air temperature, and relative humidity measurements were consistently taken at a height of 5 m for standardization.

The four radiation components exhibit oscillatory variations, with an overall decreasing trend in magnitude during the observation period. Since the three sites are relatively close in spatial proximity and DSR is primarily derived from solar radiation^51^, the DSR values and trends at the three sites are highly similar (Fig. 6a). In contrast, USR is predominantly influenced by surface albedo, which is higher in desert regions than in oases and wetlands^52^. As a result, although the USR trends are consistent, the average USR at the Huazhaizi site is 37.3% and 28.3% higher than those at the Daman and Wetland sites, respectively (Fig. 6b). For DLR, which originates mainly from thermal emissions by atmospheric water vapor and carbon dioxide^53^, the more humid conditions at the Daman and Wetland sites result in higher average DLR values, exceeding those at the arid Huazhaizi site by 7.9% and 7.5%, respectively (Fig. 6c). ULR, closely related to surface properties^54^, tends to be higher in desert regions due to elevated LST during the day and non-precipitation periods. Consequently, the average ULR at the Huazhaizi site is 1.2% and 1.4% higher than those at the Daman and Wetland sites, respectively (Fig. 6d).

Although the wind speed trends are similar across the sites, their magnitudes differ significantly. During the observation period, the average wind speed at the Daman site was the lowest (1.46 m/s), followed by the Wetland site (2.03 m/s), while the Huazhaizi site had the highest value (3.27 m/s). These differences are strongly linked to land cover types: the Daman site, with crops, buildings, and shelterbelts, experiences significant wind blockage; the Huazhaizi site, characterized by barren land, faces minimal obstruction; and the Wetland site, with buildings and vegetation but lacking tall trees, exhibits intermediate wind speeds.

Air temperature at all three sites followed similar trends, consistent with the seasonal transition from summer to winter. The average air temperature at the Huazhaizi site was 3.23 °C and 2.24 °C higher than those at the Daman and Wetland sites, respectively. Relative humidity, inversely correlated with temperature, was highest at the Daman site (61.35%), lowest at the Huazhaizi site (40.86%), and intermediate at the Wetland site (47.10%). Air pressure showed relatively small variations over the observation period but was inversely related to site elevation, decreasing with increasing altitude.

In summary, the oasis region (Daman) is characterized by humid and cool conditions, the desert region (Huazhaizi) by warm and dry conditions, and the wetland region (Wetland) exhibits intermediate characteristics. These differences highlight the distinct microclimatic features of the three sites, which are closely tied to their land cover and environmental conditions.

**Fig. 7:**
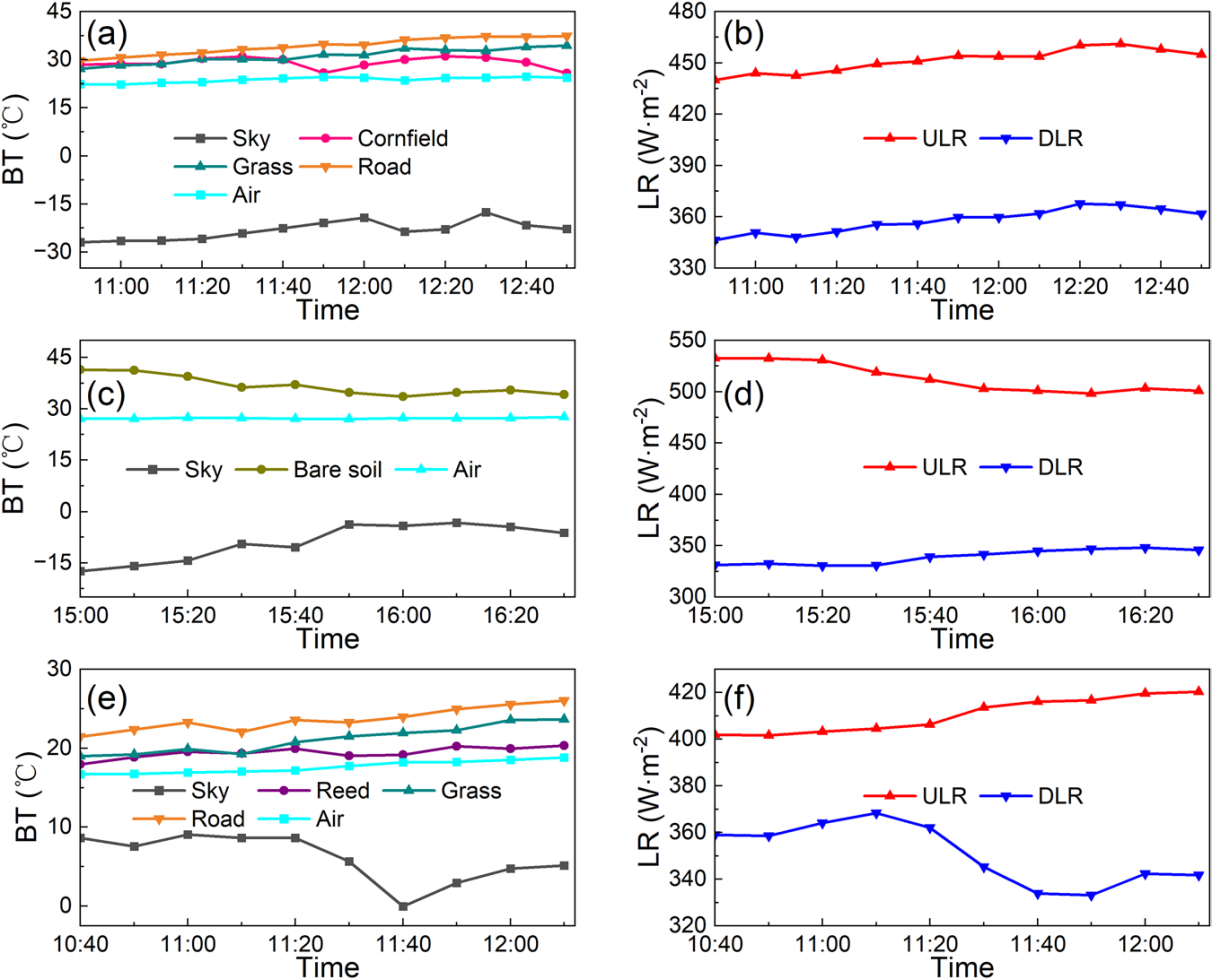
BT for typical land cover types and sky, air temperature, and longwave radiation (LR) at the three sites in September 2020. The BT for typical land cover types and sky were obtained from SI-111 TIR radiometers, while air temperature and LR were measured by automatic weather stations. (**a,b**) present data from the Daman site on September 19, 2020; (**c,d**) show data from the Huazhaizi site on September 19, 2020; and (**e,f**) display data from the Wetland site on September 20, 2020.

Taking the data from September 2020 as an example, we present the variations in BT observed by ground-based TIR radiometers at the three sites during the flight experiments. For reference, the corresponding air temperature, ULR, and DLR observed by automatic weather stations are also provided (Fig. 7). Overall, the BT of the sky is significantly lower than that of typical land cover types, and its trend closely resembles that of DLR, as both essentially characterize the thermal radiation emitted by atmospheric components such as water vapor and carbon dioxide. Additionally, the air temperature exhibits minimal variation, with fluctuations of less than 1 °C throughout the observation period. Specifically, the standard deviations of air temperature are 0.83 °C at the Daman site, 0.16 °C at the Huazhaizi site, and 0.75 °C at the Wetland site. The BT of land cover types shows an increasing trend in the morning (Fig. 7a,e) and a decreasing trend in the afternoon (Fig. 7c). These trends align with those of ULR, as ULR directly reflects the thermal radiation emitted by the surface, which is closely related to surface BT.

At the Daman site, the BT of the road is the highest (average: 34.24 °C), followed by grassland (average: 31.13 °C) and cornfield (average: 29.08 °C). Notably, the BT of the cornfield experiences a sudden drop around 11:50, likely due to obstruction by local cloud cover (Fig. 7a). Furthermore, both DLR and ULR exhibit a gradual increasing trend during the observation period (Fig. 7b). Although the observation periods at the Daman and Huazhaizi sites correspond to the morning and afternoon of the same day, respectively, the BT at the Huazhaizi site is generally higher than that at the Daman site. This is evident from the fact that the average BT of bare soil at the Huazhaizi site is 5.68 °C higher than that of the road at the Daman site. Correspondingly, the ULR at the Huazhaizi site is also 61.84 W·m-² higher than that at the Daman site. At the Wetland site, the BTs of various land cover types also demonstrate good consistency, with the road having the highest (average: 23.64 °C), reeds the lowest (average: 19.44 °C), and grassland in between (average: 21.11 °C). The BT of the sky and DLR also reach a local minimum around 11:40. These results indicate strong consistency among the observational data, reflecting their reliability and stability.

## Data Availability

The datasets associated with this article, including temperature drift-corrected BT orthomosaics, multispectral orthomosaics, NDVI maps, as well as automatic weather station and ground-based TIR radiometer observations, are freely available from the National Tibetan Plateau Center. The dataset’s accession number and direct download link are as follows: 10.11888/Terre.tpdc.302412.

## Code availability

The primary data processing, including UAV image stitching and orthomosaic creation, was performed using the software Pix4D Mapper (v4.7). For data plotting and visualization, OriginPro (v2024a) was used, and for map production, ArcMap (v10.2) was employed. To enhance the reproducibility of our dataset, the core custom Python (v3.10) scripts utilized for the correction algorithms, specifically the DRAT method and the relative radiometric normalization of multispectral images, have been organized and made publicly available. These scripts, along with a detailed README file for usage instructions, are deposited in the Zenodo repository and can be accessed at 10.5281/zenodo.18869078. Additionally, minor data handling and quality control tasks were performed leveraging standard Python libraries such as gdal, rasterio, and pandas. All key parameters and methods for data processing are detailed in the “Methods” section of the manuscript.
